# Robust Deep Network with Maximum Correntropy Criterion for Seizure Detection

**DOI:** 10.1155/2014/703816

**Published:** 2014-07-06

**Authors:** Yu Qi, Yueming Wang, Jianmin Zhang, Junming Zhu, Xiaoxiang Zheng

**Affiliations:** ^1^Qiushi Academy for Advanced Studies, Zhejiang University, Hangzhou 310027, China; ^2^Department of Computer Science, Zhejiang University, Hangzhou 310027, China; ^3^Second Affiliated Hospital of Zhejiang University, College of Medicine, Hangzhou 310000, China; ^4^Department of Biomedical Engineering, Zhejiang University, Hangzhou 310027, China

## Abstract

Effective seizure detection from long-term EEG is highly important for seizure diagnosis. Existing methods usually design the feature and classifier individually, while little work has been done for the simultaneous optimization of the two parts. This work proposes a deep network to jointly learn a feature and a classifier so that they could help each other to make the whole system optimal. To deal with the challenge of the impulsive noises and outliers caused by EMG artifacts in EEG signals, we formulate a robust stacked autoencoder (R-SAE) as a part of the network to learn an effective feature. In R-SAE, the maximum correntropy criterion (MCC) is proposed to reduce the effect of noise/outliers. Unlike the mean square error (MSE), the output of the new kernel MCC increases more slowly than that of MSE when the input goes away from the center. Thus, the effect of those noises/outliers positioned far away from the center can be suppressed. The proposed method is evaluated on six patients of 33.6 hours of scalp EEG data. Our method achieves a sensitivity of 100% and a specificity of 99%, which is promising for clinical applications.

## 1. Introduction

Epilepsy is a common and serious brain disorder, which affects about 50 million people worldwide [1]. Epileptic seizures are characterized by convulsions, loss of consciousness, and muscle spasms resulting from excessive synchronization of neuronal activities in the brain [2]. The abnormal neuronal discharges lead to epileptic patterns such as closely spaced spikes and slow waves in electroencephalogram (EEG). In seizure diagnosis and evaluation, visual inspection of these epileptic patterns from long-term EEG is a routine job for the doctors, which could be highly tedious and time-consuming [3]. Therefore, reliable seizure detection system that identifies seizure events automatically would facilitate seizure diagnosis and has great potential in clinical applications.

There are two key points in automatic seizure detection. One is how to capture the diverse patterns of seizure EEG. For different individuals, the morphologies of seizure patterns could vary considerably. Therefore, effective feature extraction plays a key role in seizure detection and lots of efforts have been made. In order to characterize the changes in amplitude and energy in epileptic EEG, Saab and Gotman [4] proposed to use three measures, relative average amplitude, relative scale energy, and coefficient of variation of amplitude. Similarly, Majumdar and Vardhan [5] utilized the variance of differentiation of time window to detect significant changes in EEG signals. To identify the sharp waves which typically appear in seizure signals, Yadav et al. [6] introduced a morphology-based detector based on the slopes of the half-waves of signals. To characterize the intrinsic time-frequency components of seizure patterns, Ghosh-Dastidar et al. [7] used principal component analysis and Zandi et al. [8] applied wavelet transform to decompose the EEG signal for feature enhancement. To encode the changes in dynamics of epileptic signal, Jouny and Bergey [9] utilized nonlinear measures of sample entropy and Lempel-Ziv complexity. To describe the topology state of epilepsy, Santaniello et al. [10] transformed the multichannel EEG data into a cross-power matrix, and eigenvalues of the matrix are used for seizure detection. The other key point is how to reduce the effect of noise. The noises caused by electromyography (EMG) or electrode movements commonly appear in EEG signal and are prone to trigger false alarms. These artifacts could bring impulsive changes with large amplitudes in EEG signal and lead to outlying values in the feature space. Some existing methods simply assumed these noises to be Gaussian [11, 12] and thus would be fragile given large amounts of outliers. Other approaches applied specific false alarm avoidance methods against these noises [4–6].

Although existing methods have shown some strengths in specific EEG datasets, the following problems have not yet been well explored. First, most existing features are designed according to the observation of a few seizure patterns, which seems too empirical to cover a wide range of seizure patterns; thus the features are usually suboptimal. Second, existing methods could be sensitive to the noises in EEG signals. Artifacts caused by EMG or electrode movements probably lead to a EEG signal shape similar to that of seizure states. A simple Gaussian assumption for the noises can be incorrect and the approaches designed based on this can cause high false alarms [11, 12]. Finally, most methods design the feature and classifier individually. Few efforts have been made to study the relationship between them or simultaneously optimize both of the two parts to maximize the abilities of them.

Inspired by the great success of deep network in image retrieval, speech recognition, and computer vision [13–21], this paper proposes a deep model framework to deal with the above issues. The main contributions of our work can be summarized as follows.

- Instead of manually designing a feature, we propose a network called robust stacked autoencoder (R-SAE) to automatically learn a feature to represent seizure patterns. The reconstruction error is first used to learn an initial feature.
- To reduce the effect of noises on EEG signals, we formulate a maximum correntropy criterion (MCC) to the R-SAE network. Unlike the traditional autoencoder model which uses the mean square error (MSE) as the reconstruction cost, the output of the new kernel MCC increases more slowly than that of MSE when the input goes away from the center. Thus, the effect of those noises/outliers positioned far away from the center can be suppressed.
- The R-SAE part and classification part are integrated to a new deep network. The objective of the network is the best seizure classification accuracy. Thus, both the initial feature and the classifier could be optimized according to the detection objective so that the whole detection system could be as optimal as possible. Besides, the optimal feature is completely data-driven. Given enough training data, the optimal feature learned by our method is able to represent various seizure patterns.

Our method is evaluated on 33.6 hours of EEG signals from six patients. With the MCC-based R-SAE model, robust features are extracted from noisy EEG signal that the sensitivity and specificity increase by 14% and 1% compared with the traditional stacked autoencoder (S-SAE). By supervised joint optimization of our deep model, the features are further optimized with better separability in the feature space and the sensitivity and specificity increase by 8% and 15%, respectively. In comparison with other methods, the proposed R-SAE model outperforms the competitors and achieves a high sensitivity of 100% and a specificity of 99%.

The rest of this paper is organized as follows.  Section 2 presents the detail of the R-SAE deep model. The experimental results and discussions are shown in Section 3. Finally, we draw the conclusions in Section 4.

## 2. Materials and Methods

**Figure 1:**
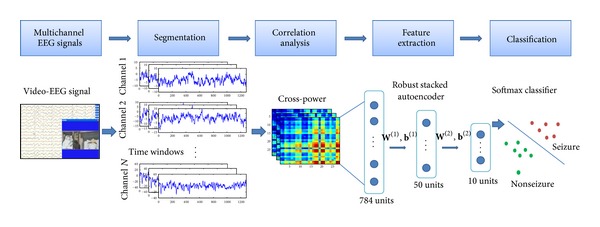
Framework of our method.

The framework of our method is shown in Figure 1. The multichannel EEG signals are firstly divided into short-time segments, and we calculate the cross-power matrix for each segment to reveal the spatial patterns of the brain. Then, compact features are extracted from the cross-power matrix by a deep network cascaded to a softmax classifier. In our method, the deep network is first pretrained with the R-SAE model to extract useful features, and then the features are further optimized jointly with the classifier to obtain optimal seizure detection system.

### 2.1. EEG Data

Scalp EEG data of six patients are used in this study. The EEG data were recorded during long-term presurgical epilepsy monitoring using NicoletOne amplifier at Second Affiliated Hospital of Zhejiang University, College of Medicine. A total of 28 channels were acquired at the sample rate of 256 Hz according to 10–20 electrode placement systems. The detail of the EEG data is given in Table 1. For each patient, all the available seizure EEG signals are used, and we randomly choose two 2.8-hour-long EEG segments as the nonseizure data segmentation and data preparation.

### 2.2. Segmentation and Data Preparation

In the preprocessing stage, the multichannel EEG data are divided into 5-second-long segments with a sliding window. For each patient, a total of 4000 segments of nonseizure data and 1000 segments of seizure data are divided from the EEG signals. There is no overlap between nonseizure segments, while, for seizure segments, the proportion of overlap is configured considering the total length of the seizure signal and number of segments required.

After segmentation, all the segments are disordered and we randomly pick 750 seizure segments and 750 nonseizure segments as the training set and the rest 3500 segments are used as the testing set. All the experiments are carried out on the same training and testing set.

### 2.3. Multichannel Analysis

Studies have shown that the correlation structure of all pairs of EEG channels could reflect the spatiotemporal evolution of electrical ictal activities [22–24]. By characterizing the spatiotemporal patterns, it is possible to identify seizures and analyze seizure dynamics.

In this study, we adopt cross-power matrix [10] to reflect the spatial patterns of the brain. For each time window with *N* channels, the cross-power matrix **A** is *N* × *N*. Each element *a*_*ij*_ in **A** is defined by the cross-power [10] between the two EEG channels *i* and *j* in a given frequency band of [*lb*, *ub*] as follows:

(1) $$\begin{array}{c} a_{ij}=\overset{ub}{\underset{lb}{\int }}P_{ij}\left(\omega \right)d\omega , \end{array}$$

where *P*_*ij*_(*ω*) is the cross-power spectral density of channels *i* and *j* at frequency *ω*.

### 2.4. Frequency Band Selection

Considering the diversity of epileptic patterns among patients, we choose the frequency band patient specifically from theta (4–7 Hz), alpha (8–13 Hz), and beta (14–30 Hz) bands. In order to select the frequency band that best reflects the difference between seizure and nonseizure states, we adopt Fisher's discriminant ratio (FDR) [25] as the criterion as follows:

(2) $$\begin{array}{c} C=\frac{{\left(\mu_{s}-\mu_{n}\right)}^{2}}{\sigma_{s}^{2}+\sigma_{n}^{2}}, \end{array}$$

where *μ*_*s*_ and *σ*_*s*_^2^ are means and covariance, respectively, of cross-power matrix of seizure segments and *μ*_*n*_ and *σ*_*n*_^2^ are those of nonseizure segments. For each patient, only the training segments are utilized for frequency band selection, and the frequency band with the highest FDR is used for seizure detection. The frequency band selected for each patient is shown in Table 1.

### 2.5. Robust Stacked Autoencoder

After multichannel analysis, each time window is represented by a cross-power matrix of *N* × *N*, where *N* denotes the number of EEG channels. We propose to employ robust stacked autoencoders to extract reliable and compact features from the cross-power matrix.

In this section, first, we briefly introduce the basic autoencoder. Then, the robust autoencoder with MCC is presented to improve the feature learning ability under noises. Finally, we stack the robust autoencoders into a deep model for compact feature extraction.

#### 2.5.1. Basic Autoencoder

Here, we begin with the traditional standard stacked autoencoder model (S-SAE). An autoencoder is a three-layer artificial network including an encoder and a decoder. The encoder takes an input vector **x** and maps it to a hidden representation **x**′ through a nonlinear function as follows:

(3) $$\begin{array}{c} x^{\prime }=s\left(W^{\left(1\right)}x+b^{\left(1\right)}\right), \end{array}$$

where *s*(·) is the sigmoid function. Suppose **x** and **x**′ are *d*-dimensional and *d*′-dimensional vectors, respectively; then **W** ^(1)^ is a *d*′ × *d* weight matrix and **b** ^(1)^ is a *d*′-dimensional bias vector.

Then, the vector **x**′ is mapped back to a reconstruction vector **y** by the decoder as follows:

(4) $$\begin{array}{c} y=s\left(W^{\left(2\right)}x^{\prime }+b^{\left(2\right)}\right), \end{array}$$

where the output vector is *d*-dimensional, **W** ^(2)^ is *d* × *d*′, and **b** ^(2)^ is a *d*-dimensional bias vector.

The parameter set *θ* = {**W** ^(1)^, **b** ^(1)^, **W** ^(2)^, **b** ^(2)^} is optimized by minimizing the average reconstruction error as follows:

(5) $$\begin{array}{c} \theta =\underset{\theta }{\text{argmin}}\frac{1}{n}\overset{n}{\underset{i=1}{\sum }}L\left(x_{i},y_{i}\right), \end{array}$$

where *L* is the loss function. Mostly, the mean square error (MSE) is used as

(6) JMSE(θ)=1n∑i=1nLMSE(xi,yi)=1n∑i=1n(12||yi−xi||2).

#### 2.5.2. Robust Autoencoder

**Figure 2:**
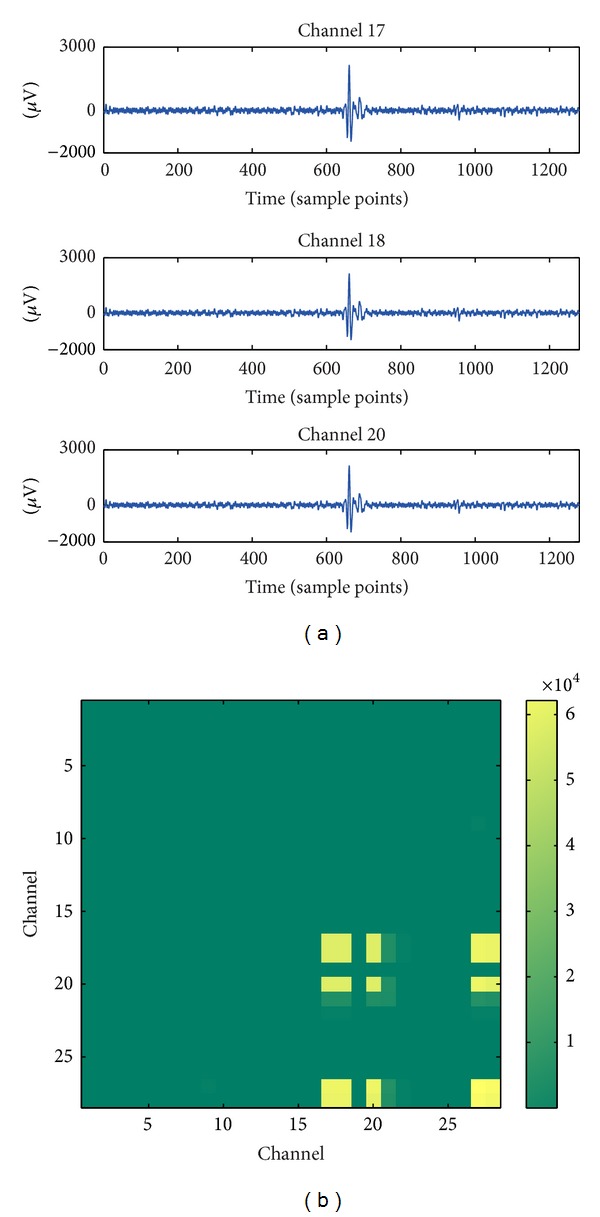
An EEG segment with impulsive noises. (a) EMG artifacts cause short-term burst noises in some channels of EEG signal; (b) visualization of the cross-power matrix of the segment with noises. The vertical and horizontal axes denote the channels and each* point *(*i*, *j*) in this figure is the cross-power value of channel *i* and channel *j*. The cross-power matrix contains outliers with large values. Because of the noise, the cross-power between channel 17 and channel 18 is far away from the interquartile range value (5.41 × 10^4^ versus 395.3).

The traditional autoencoder model based on MSE loss is not suitable for stable feature learning in EEG signals. In EEG, especially in scalp EEG signals, the large amount of noises caused by EMG artifacts or electrode movements could bring abrupt changes in EEG signal and lead to outliers in both time and frequency domain. A typical example is shown in Figure 2. In this time window, the EEG signals are noised by short-term EMG artifacts which lead to abrupt large-amplitude vibrations in some of the channels as shown in Figure 2(a). In the cross-power domain, such artifacts lead to outlying large values as in the light blocks in Figure 2(b). In the example illustrated, the cross-power between channel 17 and channel 18 is 5.41 × 10^4^, which is far away from the interquartile range value of 395.3. In this situation, the MSE-based cost of the traditional autoencoder model could be dominated by these outliers so that the feature learning ability is weakened.

In order to learn robust features from EEG signals, we replace the loss function of the autoencoder model with correntropy-based criterion to build robust autoencoder.

*Maximum Correntropy Criterion.* Correntropy is defined as a localized similarity measure [26] and it has shown good outlier suppression ability in studies [27, 28]. For two random variables *X* and *Y*, the correntropy is defined as

(7) $$\begin{array}{c} V_{\sigma }\left(X,Y\right)=E\left[\kappa_{\sigma }\left(X-Y\right)\right], \end{array}$$

where *E*[·] is the mathematical expectation and *κ*_*σ*_(·) is the Gaussian kernel with kernel size of *σ* as follows:

(8) $$\begin{array}{c} \kappa_{\sigma }\left(\cdot \right)=\frac{1}{\sqrt{2\pi }\sigma }\exp\left(-\frac{{\left(\cdot \right)}^{2}}{2\sigma^{2}}\right). \end{array}$$

**Figure 3:**
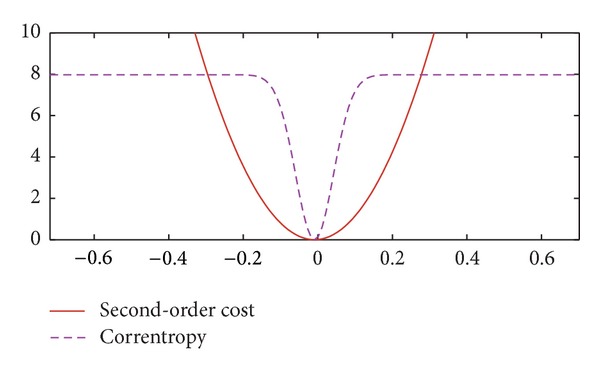
Illustration of second-order cost (red solid line) and correntropy cost (purple dashed line).

The correntropy induces a new metric that, as the distance between *X* and *Y* gets larger, the equivalent distance evolves from 2-norm to 1-norm and eventually to zero-norm when *X* and *Y* are far apart [29]. Compared with second-order statistics such as MSE, correntropy is less sensitive to outliers.  Figure 3 compares the second-order cost and correntropy cost. As the input *x* goes further from the center, the second-order cost increases sharply, so that it is sensitive to outliers. By contrast, the correntropy is only sensitive in a local range and the increase of the cost is extremely slow when the input value goes out of the central area. Therefore, the correntropy measure is particularly effective in outlier suppression.

In practice, the joint probability density function is unknown and usually only a finite set of samples of {(*x*_*i*_,*y*_*i*_)}_*i*=1_^*N*^ is available for both *X* and *Y*; then the estimated correntropy can be calculated by

(9) $$\begin{array}{c} {\tilde{V}}_{\sigma }\left(X,Y\right)=\frac{1}{N}\overset{N}{\underset{i=1}{\sum }}\kappa_{\sigma }\left(x_{i}-y_{i}\right). \end{array}$$

The maximum of correntropy error in (9) is called the maximum correntropy criterion (MCC) [29]. Due to the good outlier rejection property of correntropy, MCC is suitable for robust algorithm design.

*Robust Autoencoder Based on MCC.* In order to improve the antinoise ability of traditional autoencoders, we measure the reconstruction loss between the input vector **x** and the output vector **y** by MCC instead of MSE. In the MCC-based robust autoencoder, the cost function *J* is formulated as

(10) JMCC(θ)=1n∑i=1nLMCC(xi,yi)=1n∑i=1n ‍∑j=1mκσ(xij−yij),

where *n* is the number of training samples and *m* is the length of each training sample. The optimal parameter *θ* is obtained when *J*_MCC_(*θ*) is maximized.

In order to encourage the deep model to capture more implicit patterns, a sparsity-inducing term is adopted. Studies of sparse coding have shown that the sparseness seems to play a key role in learning useful features [30, 31]. Xie et al. [32] combined the virtues of sparse coding and deep networks into a sparse stacked denoising autoencoder to achieve better feature learning and denoising performance. In our model, we regularize the reconstruction loss by a sparsity-inducing term defined as in [32] as follows:

(11) $$\begin{array}{c} J_{\text{sparse}}\left(\theta \right)=\beta \overset{s_{2}}{\underset{i=1}{\sum }}\text{KL}\left(\rho ||{\hat{\rho }}_{i}\right), \end{array}$$

where *β* is the weight adjustment parameter, *s*_2_ is the number of units in the second layer, ${\hat{\rho }}_{i}$ is the activation value for the *i*th hidden layer unit, and *ρ* is a small number. The sparsity-inducing term constrains that the value of ${\hat{\rho }}_{i}$ should be near *ρ* under Kullback-Leibler divergence.

Also, a weight decay term *J*_weight_(*θ*) is added to avoid overfitting. It is defined as follows:

(12) $$\begin{array}{c} J_{\text{weight}}\left(\theta \right)=\frac{\lambda }{2}\overset{2}{\underset{l=1}{\sum }}\;\overset{s_{l}}{\underset{i=1}{\sum }}\;‍\overset{s_{l+1}}{\underset{j=1}{\sum }}{\left(w_{ji}^{\left(l\right)}\right)}^{2}, \end{array}$$

where *w*_*ji*_^(*l*)^ represents an element in *W* ^(*l*)^, *λ* is the parameter to adjust the weight of *J*_weight_(*θ*), and *s*_*l*_ denotes number of units in layer *l*. Therefore, the cost function of the proposed robust autoencoder is defined as

(13) $$\begin{array}{c} J_{\text{R-SAE}}\left(\theta \right)=-J_{\text{MCC}}\left(\theta \right)+J_{\text{weight}}\left(\theta \right)+J_{\text{sparse}}\left(\theta \right). \end{array}$$

By minimizing the cost of *J*_R-SAE_(*θ*), the parameter set *θ* could be optimized.

#### 2.5.3. Stacking Robust Autoencoders into Deep Network

In order to learn more effective features for seizure classification, we stack the robust autoencoders into a deep model. Stacking the robust autoencoders works in the same way as stacking the ordinary autoencoders [17] and the output from the highest layer is cascaded to a softmax classifier for seizure detection. Such a model aims at the best seizure classification accuracy, and it is able to simultaneously optimize the feature and classifier.

The training process of the deep network includes two stages: unsupervised pretraining and supervised fine-tuning. In the pretraining stage, the network is trained layer-wisely by the proposed robust autoencoder model to learn useful filters for feature extraction. A well pretrained network yields a good starting point for fine-tuning [33]. In the fine-tuning stage, a softmax classifier is added to the output of the stack, and the parameters of the whole system are tuned to minimize the classification error in a supervised manner. The network is globally tuned through back-propagation and all the parameters of both feature extraction and classification are jointly optimized. After fine-tuning, the deep network is well configured to obtain optimal overall classification performance.

## 3. Results and Discussion

In this section, experiments are carried out to evaluate the seizure detection performance of our model. The experiments include four parts: (1) we compare the unsupervised feature learning performance of the modified R-SAE model and the standard stacked autoencoder (S-SAE); (2) we compare the features before and after supervised fine-tuning to demonstrate the strength of joint optimization; (3) we compare the seizure detection performance of R-SAE model with other methods; (4) we evaluate the influence of parameters in the R-SAE model on the seizure detection performance.

In our experiments, the seizure detection performance is evaluated with the two commonly used criteria, sensitivity and specificity. Sensitivity is defined as the percentage of true seizure segments detected and specificity is the proportion of nonseizure segments correctly classified.

### 3.1. Performance of Feature Learning

**Figure 4:**
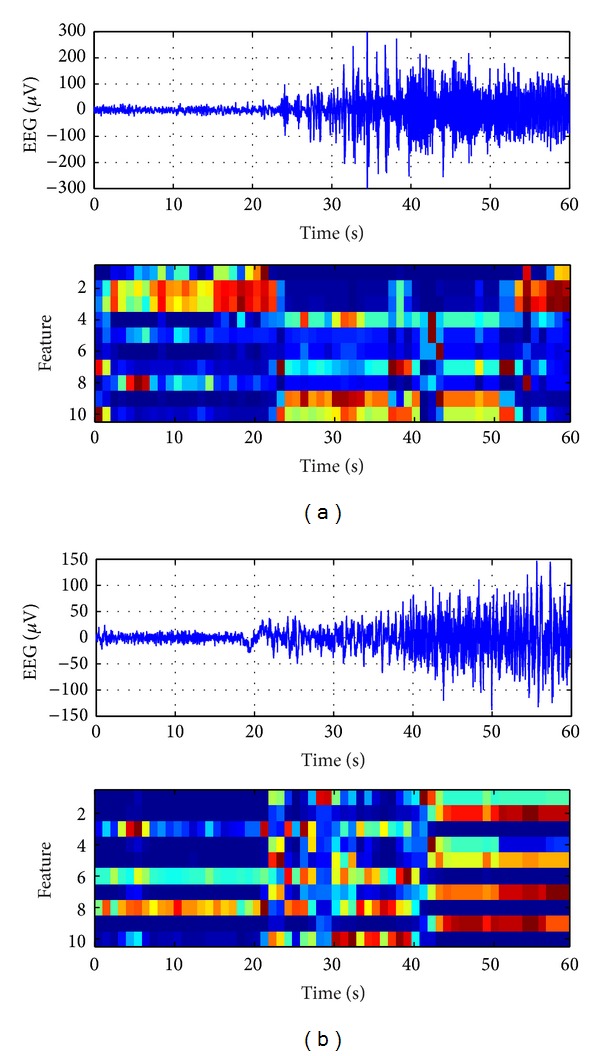
Unsupervised feature learning results by R-SAE model for patient pt03 (a) and pt04 (b). For each subfigure, the top is the original EEG signal from one channel and the bottom is the features extracted by the R-SAE model.

In this experiment, we evaluate the unsupervised feature learning ability of the R-SAE model with EEG signals. In our method, we train the R-SAE model to learn compact features from the cross-power matrix. After the layer-wised self-taught training, the deep network is well configured to learn useful features. The feature extraction results of the proposed R-SAE model are illustrated in Figure 4. For both illustrations, the seizure begins at about the 20th second. After seizure onset, the patterns of features extracted by R-SAE model show clear differences from nonseizure ones.

The feature learning performance of R-SAE and S-SAE is compared using EEG signal. In order to evaluate the ability of the features quantitatively, we utilize the classification performance as the criterion. In this experiment, the cost function of the S-SAE model is as follows:

(14) $$\begin{array}{c} J_{\text{S-SAE}}\left(\theta \right)=J_{\text{MSE}}\left(\theta \right)+J_{\text{weight}}\left(\theta \right)+J_{\text{sparse}}\left(\theta \right), \end{array}$$

where the loss function *J*_MSE_(*θ*) is formulated with MSE-based loss function as in (6) and *J*_weight_(*θ*) and *J*_sparse_(*θ*) are formulated the same as R-SAE.

We stack two autoencoders to constitute a three-layer network with 784 input units, 50 hidden units, and 10 output units. The same stacked architectures are applied for both R-SAE and S-SAE. The networks are initialized randomly and trained layer-wisely using back-propagation to minimize the cost functions. The parameters are set as *λ* = 0.003, *β* = 3, and *ρ* = 0.1 for both methods and *σ* = 0.05 for R-SAE.

The seizure detection results of both R-SAE model and S-SAE model are shown in Table 2. In order to eliminate the effects of randomness in network initialization, we present all the results averaged over 10 trials. Results show that the average sensitivity of R-SAE is 97%, which demonstrates 14% improvement compared with S-SAE. With specificity, the average result is 92% for R-SAE which is also higher than that of S-SAE. Thus, R-SAE outperforms S-SAE in both sensitivity and specificity.

In the analysis of the detection results, we find that S-SAE fails mostly on EEG segments with impulsive noises such as the segment illustrated in Figure 2. Since such abrupt artifacts could appear frequently in EEG signals, the S-SAE model could not be well trained because the MSE-based cost could be dominated by the large outliers. Thus, these EEG segments could not be well represented by the S-SAE model. By contrast, the MCC in the R-SAE model is more robust to large outliers. Therefore, the proposed R-SAE method could handle noises in EEG signal well, and it provides more robust feature extraction performance than S-SAE.

### 3.2. Performance of Joint Feature Optimization

In this experiment, we test the effects of joint feature optimization. After the MCC-based unsupervised learning, the deep network is well configured to extract useful features from EEG signals. On this basis, the deep model is fine-tuned through back-propagation to jointly optimize both feature and classifier, so that the optimal overall classification performance could be achieved. In this experiment, the parameters of R-SAE are set the same as in Section 3.1 that only the unit number of the output layer is set to 3 for visualization convenience.

**Figure 5:**
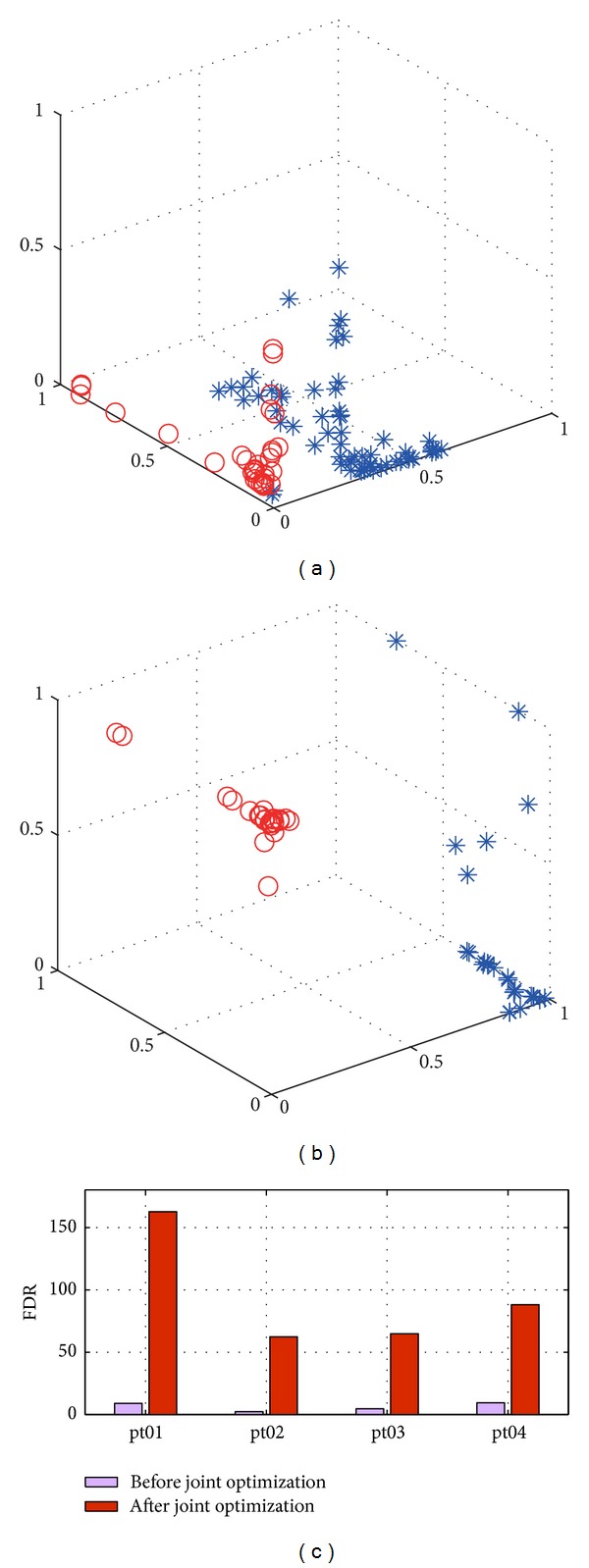
Comparison between features before and after joint optimization. (a-b) Visualization of features for seizure and nonseizure segments. The red circles denote features of seizure segments while the blue stars are nonseizure ones. (c) The FDR value of features before and after joint optimization.

The visual comparison of features before and after fine-tuning is illustrated in Figure 5. In Figures 5(a) and 5(b), the red circles denote features of seizure segments while the blue stars are nonseizure ones. It can be seen that, after fine-tuning, the seizure and nonseizure segments are more separable in the feature space. We quantitatively analyze the separability of the features before and after fine-tuning with the FDR criterion as in (2) using the first four patients. As illustrated in Figure 5(c), the fine-tuned features achieve about ten times higher FDR than do the original ones, which strongly indicates that the joint optimization could help to learn superior features with high separability, so that the seizure detection performance could be improved.

The seizure detection performance of features before and after fine-tuning is presented in Table 3. After joint feature learning, the average sensitivity of six patients increases by 8% and the specificity increases by 15%. Therefore, the joint learning process enhances the separability of features between the two classes and greatly facilitates seizure detection performance.

### 3.3. Performance of Seizure Detection

In this experiment, seizure detection performance of the proposed R-SAE model is evaluated and compared with singular value decomposition- (SVD-) based method. The SVD method is the most popular tool for correlation matrix analysis. Studies have shown that the seizure EEG signals commonly lead to a lower-complexity state which could be well reflected by the eigenvalues from SVD of the correlation matrix [10, 22].

To provide a benchmark for the comparison, we also test the seizure detection performance with the original cross-power matrix without further feature extraction. The methods included in the comparison are configured as follows.

- *SVM*: in SVM, the cross-power matrices of time windows are reshaped to vectors and fed into an SVM classifier with RBF kernel. The parameters of the SVM model are selected using 3-fold cross-validation.
- *SVD(p) + SVM*: for each time window, the cross-power matrix is decomposed by SVD, and the first *p* eigenvalues are adopted as the features. The feature vectors are then classified by an SVM classifier with RBF kernel. The parameters of the SVM model are selected using 3-fold cross-validation.
- *R-SAE(q)*: the R-SAE model is configured with 784 input units, 50 hidden units, and *q* output units. The parameters are set as *λ* = 0.003, *β* = 3, *ρ* = 0.1, and *σ* = 0.05. For this method, all results are averaged over 10 trials.

The seizure detection results of the three methods are given in Table 4. For both SVD + SVM and R-SAE, we test the seizure detection performance under two different choices of parameters of *p* and *q*, respectively. Results show that, with the original cross-power matrix classified by SVM, high sensitivities of above 0.99 are achieved for all six patients and the average specificity is 0.91. By the SVD + SVM method with *p* = 3, uneven performance is shown in different patients. For pt03, high sensitivity of 0.96 is reached with 0.99 of specificity. However, low sensitivities are obtained for pt01, pt05, and pt06. For SVD + SVM method with *p* = 10 where more features are preserved, better sensitivities and specificities are achieved. However, the uneven performance over patients still exists, and the average sensitivity is only 0.83. Since the feature extraction process of the SVD-based method loses much useful information, lower performance is obtained compared with SVM benchmark. Besides, the seizure detection performance sees a decrease when fewer eigenvalues are used. By contrast, the proposed R-SAE method achieves better performance than the benchmark SVM method. In R-SAE with *q* = 10, high sensitivities of 1.00 and specificities of 0.99 are achieved for all patients. Equally high performance is obtained with *p* = 3. The R-SAE model keeps robust seizure detection ability even with such small dimension of features.

### 3.4. Model Analysis

In this experiment, we test the influence of the two important parameters on the seizure detection performance. The first parameter is the output feature number, that is, the number of units of the output layer of the R-SAE model, and the second parameter is the kernel size *σ* in MCC. The experiment is carried out using the first four patients.

#### 3.4.1. Analysis of Feature Number

**Figure 6:**
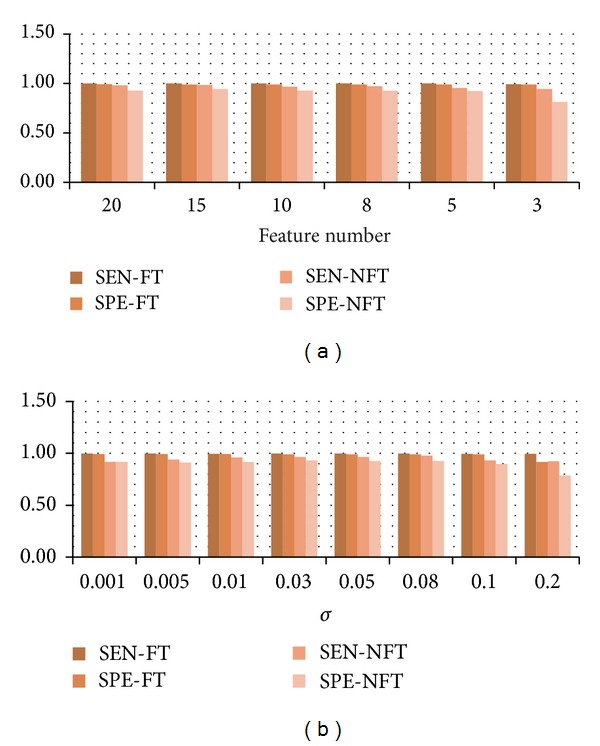
Model analysis of two important parameters of R-SAE. (a) Seizure detection performance under different feature numbers; (b) seizure detection performance with different selections of *σ*. In this figure, SEN-FT and SPE-FT are sensitivity and specificity after fine-tuning and SEN-NFT and SPE-NFT are those before fine-tuning.

The feature number is tuned by the parameter *q* in Section 3.3. In order to test the influence of *q* on seizure detection, all the other parameters are fixed as in Section 3.3 and we gradually tune *q* from 20 to 3.  Figure 6(a) illustrates the seizure detection results averaged over four patients under different choices of *q*. The result shows that the seizure detection performance of R-SAE before fine-tuning sees a slight decrease with the decrease of feature number. However, after the fine-tuning, the seizure detection performance is greatly enhanced that high sensitivities and specificities up to 99% are achieved even with small feature numbers.

#### 3.4.2. Analysis of *σ*

In the MCC, the kernel size *σ* serves as an important parameter that an appropriate choice of *σ* can effectively suppress the outliers and noises. The kernel size or bandwidth is a free parameter that its selection is still an open issue in ITL [26, 29, 34]. In practice, the parameter *σ* can be selected with Silverman's rule [35]. In the experiments of Sections 3.1–3.3, we simply set *σ* = 0.05.

Here, we test the influence of parameter *σ* on overall seizure detection performance. Also, all the other parameters are fixed as in Section 3.3.  Figure 6(b) illustrates the seizure detection results under different selections of *σ* averaged over four patients. Results show that high seizure detection performance could be achieved under a wide choice of *σ*. Better results are obtained with small *σ*, and when *σ* increases from 0.1 to 0.2, the seizure detection performance becomes worse. In practice, the choice of *σ* should be small to keep good local property of the MCC.

## 4. Conclusions

In this paper, we have presented a novel deep model which is capable of extracting robust features under large amounts of outliers. Experimental results show that the proposed R-SAE model could learn effective features in EEG signals for high performance seizure detection, and it is promising for clinical applications.

## Figures and Tables

**Table 1:** Patient information and selected frequency bands.

Patient	Sex	Chan. #	Sei. #	Hours	Freq. band
Pt01	Female	28	2	5.6	14–30 Hz
Pt02	Female	28	2	5.6	8–13 Hz
Pt03	Female	28	3	5.6	4–7 Hz
Pt04	Male	28	3	5.6	14–30 Hz
Pt05	Male	28	3	5.6	8–13 Hz
Pt06	Male	28	3	5.6	8–13 Hz

**Table 2:** Comparison between R-SAE and S-SAE (before fine-tuning).

Patient	R-SAE		S-SAE	
	Sensitivity	Specificity	Sensitivity	Specificity
Pt01	0.99 ± 8.1 × 10^−3^	0.96 ± 2.0 × 10^−2^	0.83 ± 2.0 × 10^−1^	0.92 ± 3.2 × 10^−2^
Pt02	0.96 ± 2.0 × 10^−2^	0.91 ± 1.7 × 10^−2^	0.90 ± 1.0 × 10^−1^	0.83 ± 7.6 × 10^−2^
Pt03	0.96 ± 1.5 × 10^−2^	0.93 ± 2.1 × 10^−2^	0.82 ± 2.8 × 10^−1^	0.93 ± 3.2 × 10^−2^
Pt04	0.95 ± 3.7 × 10^−2^	0.91 ± 1.3 × 10^−2^	0.91 ± 4.1 × 10^−2^	0.92 ± 2.6 × 10^−2^
Pt05	0.98 ± 1.7 × 10^−2^	0.94 ± 1.5 × 10^−2^	0.70 ± 1.3 × 10^−1^	0.96 ± 2.4 × 10^−2^
Pt06	0.97 ± 2.9 × 10^−2^	0.84 ± 5.5 × 10^−2^	0.82 ± 1.7 × 10^−1^	0.90 ± 1.5 × 10^−2^

Avg.	0.97 ± 1.3 × 10^−2^	0.92 ± 3.8 × 10^−2^	0.83 ± 6.9 × 10^−2^	0.91 ± 4.0 × 10^−2^

**Table 3:** Comparison of seizure detection performance before and after fine-tuning (FT).

Feature	Sensitivity	Specificity
Before FT	0.90 ± 1.0 × 10^−1^	0.84 ± 1.4 × 10^−1^
After FT	0.98 ± 3.1 × 10^−2^	0.99 ± 4.7 × 10^−3^

**Table 4:** Comparison with other methods.

Method	Pt01		Pt02		Pt03		Pt04		Pt05		Pt06		Avg	
	SEN∗	SPE∗	SEN	SPE	SEN	SPE	SEN	SPE	SEN	SPE	SEN	SPE	SEN	SPE
SVM	1.00	0.96	1.00	0.89	1.00	0.93	0.99	0.95	1.00	0.96	1.00	0.78	**1.00**	0.91
SVD(3) + SVM [10]	0.45	1.00	0.72	0.99	0.96	0.99	0.84	0.95	0.46	0.98	0.64	0.97	0.68	0.98
SVD(10) + SVM [10]	0.61	1.00	0.76	0.99	0.99	1.00	0.80	0.95	0.84	0.93	0.95	0.96	0.83	0.97
R-SAE(3)	1.00	0.99	0.99	0.99	0.98	0.99	1.00	0.99	1.00	0.99	0.92	0.98	0.98	**0.99**
R-SAE(10) (ours)	1.00	0.99	1.00	0.99	1.00	0.99	1.00	0.99	1.00	0.99	0.99	0.97	**1.00**	**0.99**

*SEN indicates sensitivity and SPE is specificity.
